# Expression of Concern: Development of innovative alkali activated paste reinforced with polyethylene fibers for concrete crack repair

**DOI:** 10.1371/journal.pone.0350239

**Published:** 2026-05-27

**Authors:** The PLOS One Editors

After this article [1] was published, the *PLOS One* Editors identified concerns about authorship.

In light of these concerns, the *PLOS One* Editors issue this Expression of Concern. Readers are advised to interpret the article [1] with caution.
